# Acute (*R,S*)-Ketamine Administration Induces Sex-Specific Behavioral Effects in Adolescent but Not Aged Mice

**DOI:** 10.3389/fnins.2022.852010

**Published:** 2022-04-21

**Authors:** Alessia Mastrodonato, Ina Pavlova, Noelle Kee, Josephine C. McGowan, J. John Mann, Christine A. Denny

**Affiliations:** ^1^Division of Systems Neuroscience, Research Foundation for Mental Hygiene, Inc. (RFMH)/New York State Psychiatric Institute (NYSPI), New York, NY, United States; ^2^Department of Psychiatry, Columbia University Irving Medical Center (CUIMC), New York, NY, United States; ^3^Barnard College, New York, NY, United States; ^4^Neurobiology and Behavior (NB&B) Graduate Program, Columbia University, New York, NY, United States; ^5^Molecular Imaging and the Neuropathology Division/Department of Psychiatry, Columbia University Irving Medical Center (CUIMC), New York, NY, United States

**Keywords:** adolescence, aging, behavioral despair, contextual fear discrimination, ketamine

## Abstract

(*R*,*S*)-ketamine is an *N*-methyl-D-aspartate (NMDA) receptor antagonist that was originally developed as an anesthetic. Most recently, (*R*,*S*)-ketamine has been used as a rapid-acting antidepressant, and we have reported that (*R*,*S*)-ketamine can also be a prophylactic against stress in adult mice. However, most pre-clinical studies have been performed in adult mice. It is still unknown how an acute (*R*,*S*)-ketamine injection influences behavior across the lifespan (e.g., to adolescent or aged populations). Here, we administered saline or (*R*,*S*)-ketamine at varying doses to adolescent (5-week-old) and aged (24-month-old) 129S6/SvEv mice of both sexes. One hour later, behavioral despair, avoidance, locomotion, perseverative behavior, or contextual fear discrimination (CFD) was assessed. A separate cohort of mice was sacrificed 1 h following saline or (*R*,*S*)-ketamine administration. Brains were processed to quantify the marker of inflammation Cyclooxygenase 2 (Cox-2) expression to determine whether the acute effects of (*R*,*S*)-ketamine were partially mediated by changes in brain inflammation. Our findings show that (*R,S*)-ketamine reduced behavioral despair and perseverative behavior in adolescent female, but not male, mice and facilitated CFD in both sexes at specific doses. (*R,S*)-ketamine reduced Cox-2 expression specifically in ventral CA3 (vCA3) of male mice. Notably, (*R*,*S*)-ketamine was not effective in aged mice. These results underscore the need for sex- and age-specific approaches to test (*R,S*)-ketamine efficacy across the lifespan.

## Introduction

(*R,S*)-ketamine, an *N*-methyl-D-aspartate (NMDA) receptor antagonist, was originally developed as an anesthetic and safer alternative to phencyclidine (PCP). It is still used for short-term procedural sedation in the emergency department setting in a wide age range (Rosenbaum et al., 2021). Most recently, (*R,S*)-ketamine, has emerged as a rapid-acting and long-lasting antidepressant and as a prophylactic against stress in both humans (Berman et al., 2000; Zarate et al., 2006; Murrough et al., 2013; Ma et al., 2019) and mice (Kavalali and Monteggia, 2015; Altemus et al., 2014; Amat et al., 2016; Brachman et al., 2016; Zanos et al., 2016; McGowan et al., 2017; Dolzani et al., 2018). In contrast to traditional antidepressants which take weeks to reach efficacy and require daily administration to be effective, (*R,S*)-ketamine acts within 2 h of administration (Murrough et al., 2013) in patients with major depressive disorder (MDD). Morever (*R,S*)-ketamine effects can last up to 2 weeks following injection (Zarate et al., 2006; Murrough et al., 2013).

Despite the prevalence of (*R,S*)-ketamine among children as an anesthetic (Dolansky et al., 2008; Dwyer et al., 2017), few studies have examined whether (*R,S*)-ketamine is effective in treating adolescent mood disorders (Papolos et al., 2013; Dwyer et al., 2017; Cullen et al., 2018; Zarrinnegar et al., 2019). Similarly, (*R,S*)-ketamine is used as an anesthetic in older adults (Wickström et al., 1982; Maneglia and Cousin, 1988), often combined with other drugs (Willman and Andolfatto, 2007; Andolfatto et al., 2012). However, there are few data about (*R,S*)-ketamine efficacy as an antidepressant in elderly patients (i.e., > 60 years old) (Szymkowicz et al., 2014; Heard et al., 2017; Medeiros da Frota Ribeiro and Riva-Posse, 2017; Bahr et al., 2019; Bryant et al., 2019; Pennybaker et al., 2021), and there are no preclinical studies on (*R,S*)-ketamine’s antidepressant effects in aged mice. Two studies in elderly patients showed that (*R,S*)-ketamine successfully reduced depressive symptomatology (Srivastava et al., 2015; Medeiros da Frota Ribeiro and Riva-Posse, 2017), while two other studies showed that (*R,S*)-ketamine was effective immediately after the injection, but not over time, suggesting that (*R,S*)-ketamine has low efficacy in elderly patients (George et al., 2017; Bryant et al., 2019). However, in these studies, (*R,S*)-ketamine was often given on top of other antidepressants, in patients with co-morbid disorders that weren’t statistically controlled for, and without taking into consideration sex differences. Importantly, the aforementioned studies have shown that (*R*,*S*)*-*ketamine is effective within 1 h of administration in both adolescent and aged subjects (Papolos et al., 2013; Srivastava et al., 2015) and does not induce any psychotomimetic effects 1 h post-administration (Papolos et al., 2013; Srivastava et al., 2015). Therefore, 1 h could be the optimal time point for testing (*R,S*)-ketamine efficacy in adolescent or aged subjects.

Recently, inflammatory mediators, such as cytokines, enzymes, and metabolites levels, have been used as biomarkers for depressive behavior (Wright et al., 2005; Haroon et al., 2012; Strawbridge et al., 2017). In particular, Cox-2, an enzyme that mediates inflammation by synthesizing prostaglandins, is increased in depressed patients (Galecki et al., 2012). Interestingly, (*R,S*)-ketamine has been shown to have anti-inflammatory properties in depressed patients with peripheral inflammation (De Kock et al., 2013; Zanos et al., 2018; Verdonk et al., 2019). While the potential of (*R,S*)-ketamine to produce anti-inflammatory actions has been shown (Yli-Hankala et al., 1992; Meyer and Lázaro Da Silva, 2004; Shaked et al., 2004; Gurfinkel et al., 2006; De Kock et al., 2013). None of the previous studies directly evaluate (*R,S*)-ketamine’s impact on Cox-2 expression in the brain. If (*R,S*)-ketamine changes Cox-2 expression in adolescent or aged mice is as of yet unknown.

Here, in order to better understand the acute effect of (*R,S*)-ketamine administration in adolescent and aged populations, male and female adolescent or aged mice were injected with saline or (*R,S*)-ketamine at one of varying doses. One hour later, behavioral despair, avoidance, locomotion, perseverative behavior, and CFD were assessed. (*R,S*)-ketamine attenuated behavioral despair and perseverative behaviors in adolescent female, but not male, mice and facilitated CFD in both sexes. Overall, there were no effects of (*R,S*)-ketamine in aged mice. Moreover, Cox-2 expression was increased in the hippocampus (HPC) of aged mice as compared to adolescent mice. (*R,S*)-ketamine administration specifically reduced the expression of Cox-2 in ventral CA3 (vCA3) in adolescent male mice. In summary, this study showed that (*R,S*)-ketamine impacts behavior in a sex-, dose-, and age-dependent manner perhaps by differentially altering Cox-2 expression. This study will contribute to informing clinical studies on (*R,S*)-ketamine efficacy across the lifespan.

## Materials and Methods

### Mice

For adolescent studies, male and female 129S6/SvEvTac mice were purchased from Taconic (Hudson, NY, United States) at 4 weeks of age. For aged studies, 129S6/SvEvTac mice were purchased from Taconic and bred in-house. The pups were then aged to 24 months of age. Mice were housed 5 per cage in a 12-h (06:00–18:00) light-dark colony room at 22°C. Food and water were provided *ad libitum*. Behavioral tests were performed during the light phase. All experiments were approved by the Institutional Animal Care and Use Committee (IACUC) at NYSPI.

### Drugs

A single injection of saline (0.9% NaCl) or (*R,S*)-ketamine (Fort Dodge Animal Health, Fort Dodge, IA, United States) (10, 30, or 100 mg/kg) was administered at 5 weeks or 24 months of age once during the course of each experiment. All drugs were prepared in physiological saline and administered intraperitoneally (i.p.) in volumes of 0.1 cc per 10 mg body weight.

### Behavioral Assays

#### Forced Swim Test

The FST was used to assay behavioral despair as previously described (Brachman et al., 2016; Mastrodonato et al., 2020). In this test, an animal placed in a container filled with water, will first make efforts to escape but eventually will exhibit immobility, which is thought to reflect a failure of persistence in escape-directed behavior (i.e., behavioral despair). The term behavioral despair aligns with the National Institute of Mental Health (NIMH) Research Domain Criteria (RDoC). The RDocC suggest that researchers should consider multiple levels of disease complexity (e.g., genetics, behavior, or circuitry) when studying aspects of psychiatric disorders, such as depression, with the goal of identifying mechanisms which may translate to human conditions. While no single test can capture the full complexity of a human disorder, the FST in particular lacks mechanistic specificity. However, this test has been useful to study the stress-induced behavioral phenotype and dose response curves of drugs [e.g., (*R,S*)-ketamine] over time. In this study, mice were placed into clear plastic buckets 20 cm in diameter and 23 cm deep filled 2/3 of the way with 22°C water. Mice were videotaped from the side for 6 min on 2 consecutive days. Immobility time was assessed by time spent floating. Average immobility time for day 2 of the FST was calculated for min 3–6 (4 min in total).

#### Locomotor Activity Test

The LA was administered as previously described (Brachman et al., 2016; Mastrodonato et al., 2018). Briefly, motor activity was quantified in two Plexiglas open field (OF) boxes 50 cm × 50 cm (MED Associates, Georgia, VT, United States). Mice were individually placed in the center of the OF box and allowed to explore the field for 10 min. Total distance traveled was quantified by using ANY-maze behavior tracking software (ANY-maze, RRID:SCR_014289, Stoelting, Wood Dale, IL, United States).

#### Elevated Plus Maze

Elevated plus maze was performed as previously described (Brachman et al., 2016; Mastrodonato et al., 2018). Briefly, the testing was conducted in a plus-cross-shaped apparatus consisting of 4 arms, 2 open and 2 enclosed by walls, linked by a central platform at a height of 50 cm from the floor. Mice were individually placed in the center of the maze facing an open arm and could explore the maze for 5 min. The time spent in the open arms was used as an index of anxiety. Videos were scored using ANY-maze tracking software (ANY-maze, RRID:SCR_014289, Stoelting, Wood Dale, IL, United States).

#### Marble Burying

The MB assay was performed in a clean cage (10.5 in ×5.5 in) containing soft pliable Beta Chip bedding (Northeastern Products Corp, Warrensburg, NY, United States). The cage contained 16 marbles set up in four rows of four marbles across. Mice were given 30 min to explore and bury. At the end of the assay, the number of marbles buried was calculated.

#### Contextual Fear Discrimination

In the CFD assay (Sahay et al., 2011; Mastrodonato et al., 2018), mice were exposed to a 1-shock CFC training. Mice received a single 2 s foot shock of 0.75 mA after being placed in the context for 180 s and were removed 15 s following the shock (i.e., 197 s). Mice were then exposed to two contexts daily. One context was the CFC context where the mice were shocked daily (context A), and the other context was a similar, novel context without a shock (context B). For context A, mice received 1-shock CFC daily as described above. For context B, mice were placed in the CFC chamber for 180 s with no shock. Each day, time spent freezing in each context was calculated as a percentage. The contextual information is listed in Supplementary Table 1.

### Brain Processing

Mice were deeply anesthetized, and brains were processed as previously described in Denny et al. (2014), Cazzulino et al. (2016), and Pavlova et al. (2018). Brains were then frozen in optimal cutting temperature (OCT) medium and sliced into 100 μm sections using a cryostat.

### Cox-2 Immunohistochemistry

An iDISCO-based immunohistochemistry protocol was performed (Pavlova et al., 2018). Briefly, sections were washed in 1X phosphate buffered saline (PBS) in three increments of 10 min each, then dehydrated in 50% MeOH for 2.5 h. Sections were then washed in 0.2% PBS with TritonX-100 (PBST) in three increments of 10 min each and placed in blocking solution [10% normal donkey serum (NDS)/0.1% PBST] for 2 h. After blocking, sections were washed in three increments of 10 min each in 0.1% PBST. Sections were then incubated in a solution of primary antibody mouse monoclonal IgG anti-Cox-2 (1:200, Santa Cruz Biotechnology Cat# sc-376861, RRID:AB_2722522, Santa Cruz, CA, United States) in 10% NDS/0.1% PBST for 3 days at 4°C. On day 4, sections were washed in three increments of 10 min each in 1X PBS and incubated in secondary antibody solution consisting of Alexa 647 conjugated Goat Anti-Mouse IgG (1:250, Molecular Probes Cat# A-21235, RRID:AB_2535804, Waltham, MA, United States) in 10% NDS/1X PBS overnight. The next day, sections were washed in three increments of 10 min each in 1X PBS. Sections were mounted on slides and allowed to dry for approximately 20 min before adding mounting medium Fluoromount G (Electron Microscopy Sciences, Hatfield, PA, United States) and a coverslip.

### Confocal Microscopy

All samples were imaged on a confocal scanning microscope (Leica TCS SP8, Leica Microsystems Inc., Wetzlar, Germany) with two simultaneous PMT detectors, as previously described (Pavlova et al., 2018). Fluorescence from Alexa Fluor 647 was excited at 634 nm and detected at 650–700 nm. Sections were imaged with a dry Leica 20 × objective (NA 0.70, working distance 0.5 mm), with a pixel size of 1.08 × 1.08 μm^2^, a z step of 3 μm, and z-stack of 24 μm. Fields of view were stitched together to form tiled images by using an automated stage and the tiling function and algorithm of the LAS X software (Leica Application Suite X, RRID:SCR_013673, Wetzlar, Germany).

### Cell Quantification

An investigator blind to treatment manually circled the granule cell layer (GCL) of the DG or the pyramidal layer (PL) of CA3 throughout the entire rostro-caudal axis of the hippocampus (HPC) using Fiji (Fiji, RRID:SCR_002285, Dresden, Germany) (Mastrodonato et al., 2018). The mean intensity of Cox-2 expression was measured bilaterally using Fiji (Mastrodonato et al., 2018) and normalized to the area of the GCL or PL.

### Statistical Analysis

All data were analyzed using Prism 8.0 (GraphPad Prism, RRID:SCR_002798, La Jolla, CA, United States). Alpha was set to 0.05 for all analyses. Overall, the effect of Age, Time, and Drug was analyzed using an analysis of variance (ANOVA), using repeated measures where appropriate. *Post hoc* Dunnett and Sidak tests were used where appropriate. *A priori* comparisons were undertaken when there was no significant Drug x Age interactions when a main effect of Drug was statistically significant to evaluate the impact of the Drug within the adolescent or aged cohorts. Male and female mice were tested separately to avoid any possible confounding effects due to hormonal variation, therefore they were analyzed separately.

The effect of Drug on Cox-2 expression (mean intensity) was analyzed using *t*-tests. All statistical tests and *p* values are listed in Supplementary Table 2. A summary of behavioral findings is listed in Supplementary Table 3.

## Results

### Acute (*R*,*S*)-Ketamine Reduces Behavioral Despair and Perseverative Behavior in Female, but Not Male, Adolescent Mice

**FIGURE 1:**
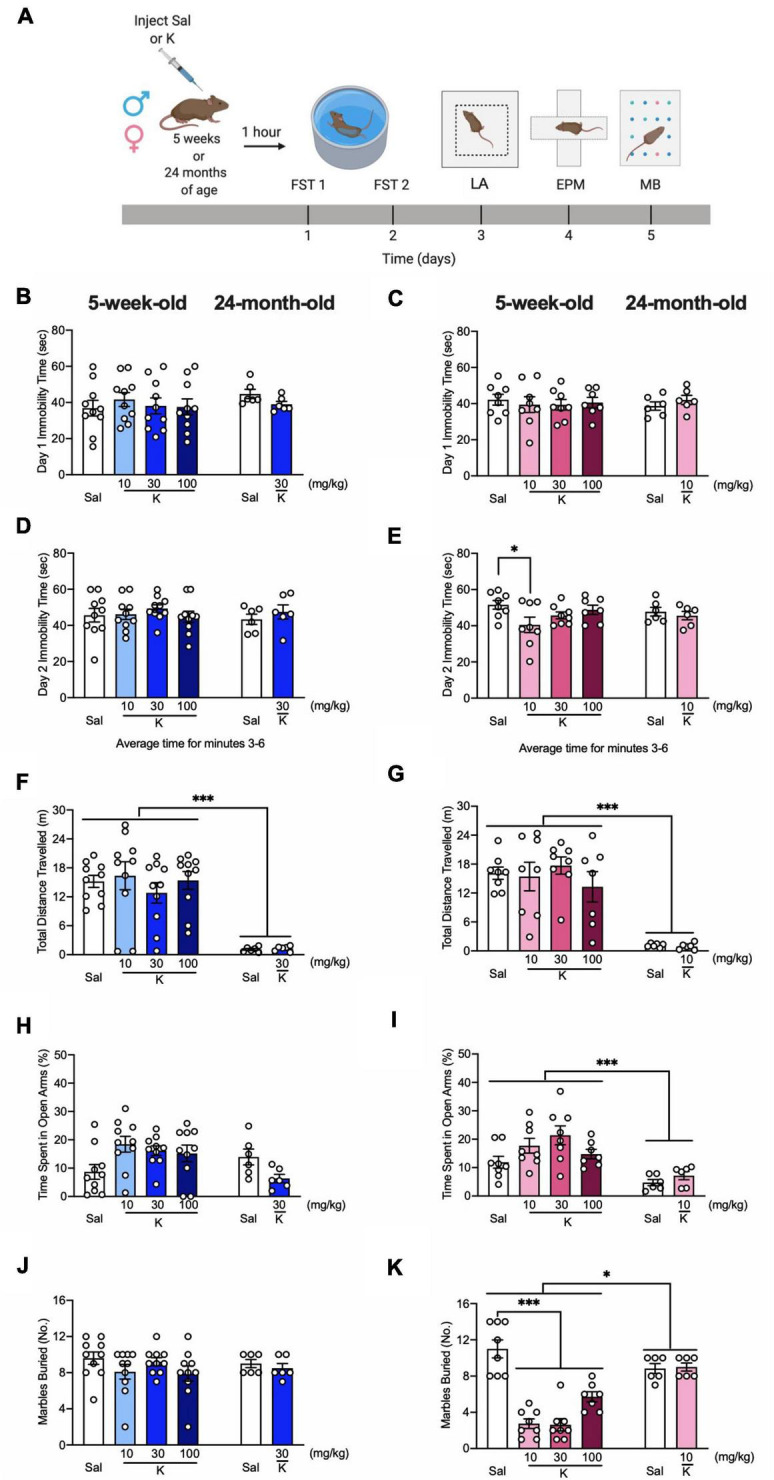
Acute (*R,S*)-ketamine administration decreases behavioral despair and perseverative behavior in adolescent female, but not male mice. **(A)** Experimental design. **(B)** Five-week-old male mice administered saline or (*R,S*)-ketamine exhibited similar immobility time to 24-month-old male mice on day 1 of FST. (*R*,*S*)-ketamine did not impact immobility time in any groups of male mice. **(C)** Five-week-old and 24-month-old female mice administered saline or (*R,S*)-ketamine exhibited similar immobility time on day 1 of FST. (*R*,*S*)-ketamine did not impact immobility time in any groups. **(D)** All groups of 5-week-old and both groups of 24-month-old male mice had comparable immobility time on day 2 of the FST. **(E)** Five-week-old female mice administered (*R*,*S*)-ketamine (10 mg/kg) exhibited reduced immobility time when compared with female mice administered saline. Both groups of 24-month-old female mice had comparable immobility time on day 2 of the FST. **(F,G)** (*R,S*)-ketamine did not affect the distance traveled in the LA in 5-week-old and 24-month old male and female mice. However, 24-four-month-old male and female mice traveled significantly less than 5-week-old mice. **(H)** Five-week- and 24-month-old male mice spent a comparable time in the open arms. **(I)** Five-week-old female mice spent a more time in the open arms than 24-month-old female mice. (*R*,*S*)-ketamine did not impact behavior in any groups. **(J)** During the MB task, all groups of male mice buried a comparable number of marbles. **(K)** All groups of 5-week-old female mice administered (*R*,*S*)-ketamine buried significantly fewer marbles when compared with female mice administered saline. Five-week-old female mice buried significantly fewer marbles than 24-month-old female mice. Both groups of 24-month-old female mice buried a comparable number of marbles. (*n* = 6–10 mice per group). Error bars represent +SEM. *p < 0.05. ^***^p < 0.0001. Sal, saline; K, (*R,S*)-ketamine; FST, forced swim test; LA, locomotor activity test; EPM, elevated plus maze; MB, marble burying; sec, second; mg, milligram; kg, kilogram; m, meter; No, number.

To determine if an acute injection of (*R*,*S*)-ketamine could impact behavior in adolescent or aged mice, male and female mice were injected with saline or (*R*,*S*)-ketamine at varying doses (Figure 1A). Doses were chosen based on previous studies in adolescent (Parise et al., 2021) and adult mice (Brachman et al., 2016; McGowan et al., 2017; Mastrodonato et al., 2018; Chen et al., 2020). Since aged mice were bred in-house, and therefore, only a limited number of mice were available for each experiment, only the previously identified doses for adult mice (30 mg/kg for males and 10 mg/kg for females) were chosen for aged experiments. One hour later, mice were assayed in the FST. During day 1 of the FST, adolescent male and female mice exhibited similar immobility time compared to aged male mice [Age: *F*_(1,48)_ = 0.888, *p* = 0.350; Age: *F*_(1,39)_ = 0.040, *p* = 0.842, respectively] (Figures 1B,C). (*R*,*S*)-ketamine did not impact immobility time in any group [Drug: *F*_(1,48)_ = 0.180, *p* = 0.673; Drug: *F*_(1,39)_ = 0.026, *p* = 0.872]. During day 2 of the FST, all groups of adolescent and aged male mice had comparable immobility time [Drug: *F*_(1,48)_ = 0.685, *p* = 0.412; Age: *F*_(1,48)_ = 0.061, *p* = 0.806] (Figure 1D). However, (*R*,*S*)-ketamine-injected female mice (10 mg/kg) exhibited decreased immobility time compared to saline-injected female mice (*p* = 0.026) (Figure 1E). (*R*,*S*)-ketamine did not alter immobility time in aged female mice (*p* = 0.528).

In the LA, adolescent mice traveled significantly more than aged mice [Drug: *F*_(1,48)_ = 0.001, *p* = 0.969; Drug: *F*_(1,39)_ = 0.039, *p* = 0.844, respectively] (Figures 1F,G). (*R*,*S*)-ketamine did not impact the distance traveled in any groups. In the EPM, adolescent and aged male mice spent a comparable time in the open arms [Drug: *F*_(1,48)_ = 0.005, *p* = 0.942; Age: *F*_(1,48)_ = 0.944, *p* = 0.336] (Figure 1H). Adolescent female mice spent significantly more time in the open arms than aged female mice [Age: *F*_(1,39)_ = 15.710; *p* = 0.0003] (Figure 1I). (*R*,*S*)-ketamine did not impact behavior in the EPM in any groups. In the MB task, adolescent and aged male mice buried a comparable number of marbles [Drug: *F*_(1,48)_ = 1.449, *p* = 0.234; Age: *F*_(1,48)_ = 0.125, *p* = 0.724] (Figure 1J). (*R*,*S*)-ketamine did not impact MB behavior in the male groups [Drug: *F*_(1,48)_ = 1.449, *p* = 0.234]. In female mice, there was a significant effect of Age on the percent of marbles buried [Drug: *F*_(1,39)_ = 23.820, *p* < 0.0001] (Figure 1K). Adolescent (*R*,*S*)-ketamine-injected mice (all doses) buried significantly fewer marbles than saline-injected mice. Both groups of aged female mice buried a comparable number of marbles. These data indicate that an acute injection of (*R*,*S*)-ketamine does not impact behavior in adolescent or aged male mice, but decreases behavioral despair and perseverative behavior in adolescent female mice.

**FIGURE 2:**
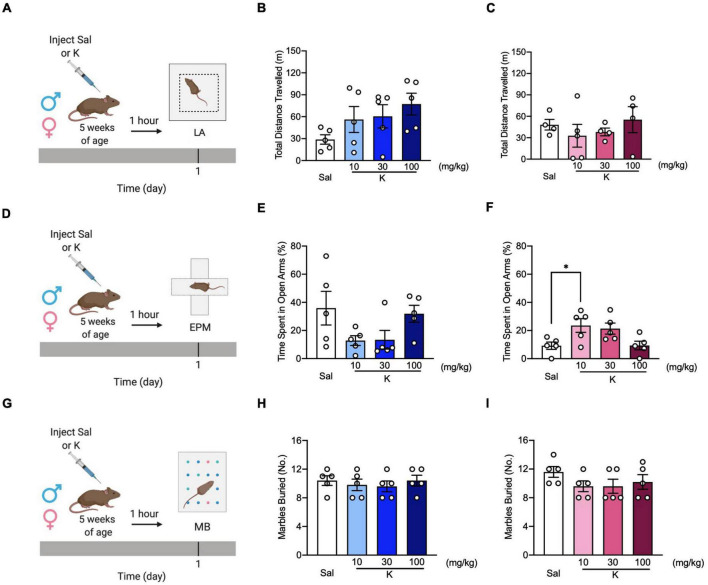
(*R,S*)-ketamine decreases avoidance behavior without affecting locomotor activity and perseverative behavior in adolescent mice. **(A)** Experimental design. **(B,C)** Male and female 5-week-old mice showed comparable distance traveled in the LA. **(D)** Experimental design. **(E)** All groups of 5-week-old male mice spent a similar time in the open arms of the EPM. **(F)** Five-week-old female mice administered (*R,S*)-ketamine (10 mg/kg) spent significantly more time in the open arms of the EPM compared to saline mice. **(G)** Experimental design. **(H,I)** Male and female of 5-week-old mice buried a comparable number of marbles. (*n* = 4–5 mice per group). Error bars represent +SEM. * *p* < 0.05. Sal, saline; K, (*R,S*)-ketamine; LA, locomotor activity test; EPM, elevated plus maze; MB, marble burying; mg, milligram; kg, kilogram; m, meter.

To investigate whether the lack of (*R,S*)-ketamine effect in some of the behavioral assays was due to time delay from drug injection, we next tested three separate cohorts of adolescent mice in the LA (Figure 2A), EPM (Figure 2D), and MB (Figure 2G) 1 h following saline or (*R,S*)-ketamine administration. Since only a limited number of aged mice were available, and (*R,S*)-ketamine was not effective in any behavioral tasks in the previous experiment (Figure 1), aged mice were not tested here. In the LA, all groups of male and female mice traveled comparably [Drug = *F*_(3,16)_ = 1.938, *p* = 0.164; Drug = *F*_(3,13)_ = 0.570, *p* = 0.644, respectively] (Figures 2B,C). In the EPM, all groups of adolescent male mice spent a comparable time in the open arms [Drug = *F*_(3,16)_ = 2.491, *p* = 0.097] (Figure 2E). However, in female mice, adolescent (*R,S*)-ketamine-injected mice (10 mg/kg) spent more time in the open arms than saline-injected mice [Drug = *F*_(3,16)_ = 4.231, *p* = 0.022] (Figure 2F). In contrast to what we observed when MB was administered 5 days following saline or (*R,S*)-ketamine administration, here, in the MB assay, adolescent male and female mice administered saline or (*R,S*)-ketamine buried a similar number of marbles [Drug = *F*_(3,16)_ = 0.306, *p* = 0.820; Drug = *F*_(3,16)_ = 1.141, *p* = 0.885, respectively] (Figures 2H,I). These data suggest that (*R,S*)-ketamine efficacy is time- and sex-dependent.

### Acute (*R*,*S*)-Ketamine Administration Facilitates Contextual Fear Discrimination in Adolescent but Not Aged Mice

**FIGURE 3:**
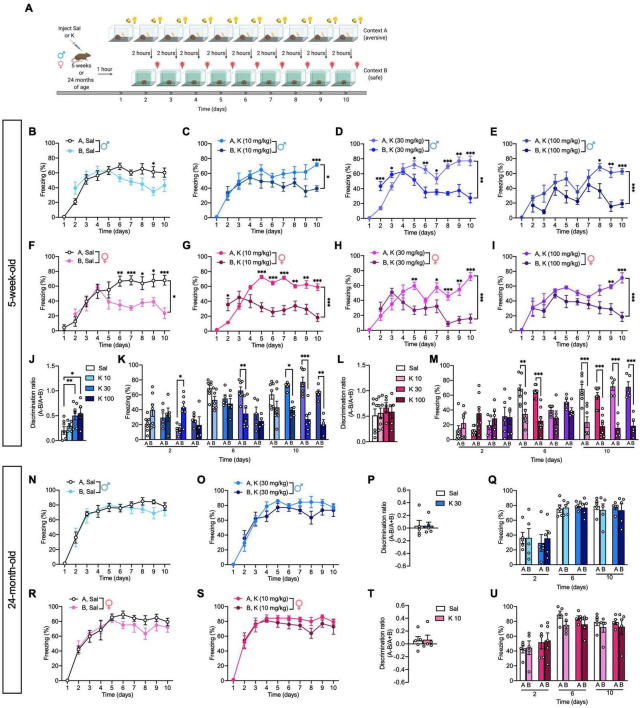
Acute (*R,S*)-ketamine facilitates contextual fear discrimination in adolescent but not aged male mice. **(A)** Experimental design. **(B)** Saline-injected male mice could not discriminate between the two contexts. **(C)** (*R*,*S*)-ketamine-injected male mice (10 mg/kg) could discriminate between the two contexts only on day 10. **(D)** (*R*,*S*)-ketamine-injected male mice (30 mg/kg) initially had increased fear generalization on day 2, but could discriminate starting on day 5. **(E)** (*R*,*S*)-ketamine-injected male mice (100 mg/kg) could discriminate between the two contexts starting from day 8. **(F)** Saline-injected female mice could discriminate between the two contexts starting on day 6. **(G,H)** (*R*,*S*)-ketamine-injected female mice (10 and 30 mg/kg) could discriminate between the two contexts starting on day 5. **(I)** (*R*,*S*)-ketamine-injected female mice (100 mg/kg) could discriminate starting on day 9. **(J)** (*R*,*S*)-ketamine-injected male mice (30 or 100 mg/kg) exhibited improved discrimination ratios on day 10. **(K)** (*R,S*)-ketamine-injected male mice (30 mg/kg) showed increased fear generalization on days 2 and 3, however, they could discriminate between the two contexts on day 6. On day 10, (*R,S*)-ketamine-injected male mice (all doses) could discriminate between the two contexts. **(L)** All groups of female mice exhibited comparable discrimination ratios on day 10. **(M)** Saline- or (*R,S*)-ketamine-injected female mice could not discriminate between the two contexts on day 2. On day 6 Saline- or (*R*,*S*)-ketamine-injected female mice (10 mg/kg) could discriminate. On day 10, all groups of mice could discriminate. **(N,O)** Twenty-four-month-old male saline- or (*R,S*)-ketamine-injected mice could not discriminate between the two contexts over the 10 days. **(P)** Both groups of male mice exhibited comparable discrimination ratios on day 10. **(Q)** On Days 2, 6, and 10, all groups exhibited comparable freezing. **(R,S)** Twenty-four-month-old saline- or (*R,S*)-ketamine-injected female mice could not discriminate between the two contexts over the 10 days. **(T)** Both groups of female mice exhibited comparable discrimination ratios on day 10. **(U)** On Days 2, 6, and 10, all groups exhibited comparable freezing. (*n* = 5–10 mice per group). Error bars represent ± SEM. * *p* < 0.05, ^**^ *p* < 0.01, ^***^ *p* < 0.0001. Sal, saline; K, (*R,S*)-ketamine; mg, milligram; kg, kilogram.

A recent study showed that a single dose of (*R,S*)-ketamine administered 22 h after CFC alleviated fear memory generalization in adult C57BL/6 mice (Asim et al., 2020). However, it remains to be determined if an acute (*R,S*)-ketamine administration could reduce fear generalization in adolescent or aged mice. Here, adolescent and aged mice were injected with saline or (*R*,*S*)-ketamine and administered a CFD paradigm 1 h later (Figure 3A). In saline mice, there was not a significant effect of Context [*F*_(1,12)_ = 2.220, *p* = 0.162], but there was a significant effect of Time [*F*_(8,96)_ = 6.161, *p* = 0.0001], and an interaction of Time x Context [*F*_(8,96)_ = 3.540, *p* = 0.001]. Unlike adult male mice (Mastrodonato et al., 2018), saline-treated adolescent male mice could only discriminate between the two contexts on day 9 (*p* = 0.010) (Figures 3B,J,K). (*R,S*)-ketamine-injected male mice (10 or 100 mg/kg) started discriminating on days 10 or 8, respectively [Context: *F*_(1,8)_ = 8.062 *p* = 0.021; Context: *F*_(1,8)_ = 28.760, *p* = 0.0007, respectively]. (Figures 3C,E,J,K). However, adolescent male mice administered (*R*,*S*)-ketamine (30 mg/kg) initially had increased fear generalization on days 2 (*p* = 0.0008) and 3 (*p* = 0.032), but discriminated starting on day 5 [Context: *F*_(1,12)_ = 14.480, *p* = 0.002] (Figures 3D,J,K). By day 10, (*R,S*)-ketamine-injected male (30 or 100 mg/kg, but not 10 mg/kg) mice showed improved discrimination ratios when compared to saline-injected male mice [Drug: *F*_(3,20)_ = 3.802, *p* = 0.026] (Figures 3J).

Interestingly, in contrast to males, there was a significant effect of Context [*F*_(1,12)_ = 8.708, *p* = 0.012], Time [*F*_(8,96)_ = 7.465, *p* < 0.0001], and an interaction of Context x Time [*F*_(8,96)_ = 5.046, *p* < 0.0001] in saline-injected female mice. Saline-injected adolescent female mice discriminated starting on day 6 (*p* = 0.005) (Figures 3F,L,M). Adolescent (*R*,*S*)-ketamine-injected female mice (10 mg/kg) initially showed increased fear generalization on day 2 (*p* = 0.014). However, (*R*,*S*)-ketamine-injected female mice (10 or 30 mg/kg) could discriminate starting on day 5 [Context: *F*_(1,14)_ = 20.680, *p* = 0.0005; Context: *F*_(1,8)_ = 30.720, *p* = 0.0005, respectively] (Figures 3G,H,L,M), (*R*,*S*)-ketamine-injected female mice (100 mg/kg) could discriminate starting on day 9 [Context: *F*_(1,8)_ = 42.130, *p* = 0.0002] (Figures 3I,L,M). By day 10, all groups of female mice showed similar discrimination ratios [Drug: *F*_(3,21)_ = 0.293, *p* = 0.830] (Figure 3L).

Notably, saline- or (*R,S*)-ketamine-injected aged mice could not discriminate between the two contexts over the 10 days of CFD testing [Context: *F*_(1,8)_ = 0.451, *p* = 0.520; Context: *F*_(1,8)_ = 1.985, *p* = 0.196, respectively] and exhibited comparable discrimination ratios on day 10 (Drug: *p* = 0.986; Drug: *p* = 0.899, respectively) (Figures 3N–U). These data are in accord with prior studies showing impaired CFD in aged mice (Wu et al., 2015), and indicate that dose-specific, acute (*R*,*S*)-ketamine administration is effective at facilitating CFD in adolescent, but not aged mice.

### (*R*,*S*)-Ketamine Decreases Cox-2 Expression in vCA3 of Adolescent but Not Aged Mice

**FIGURE 4:**
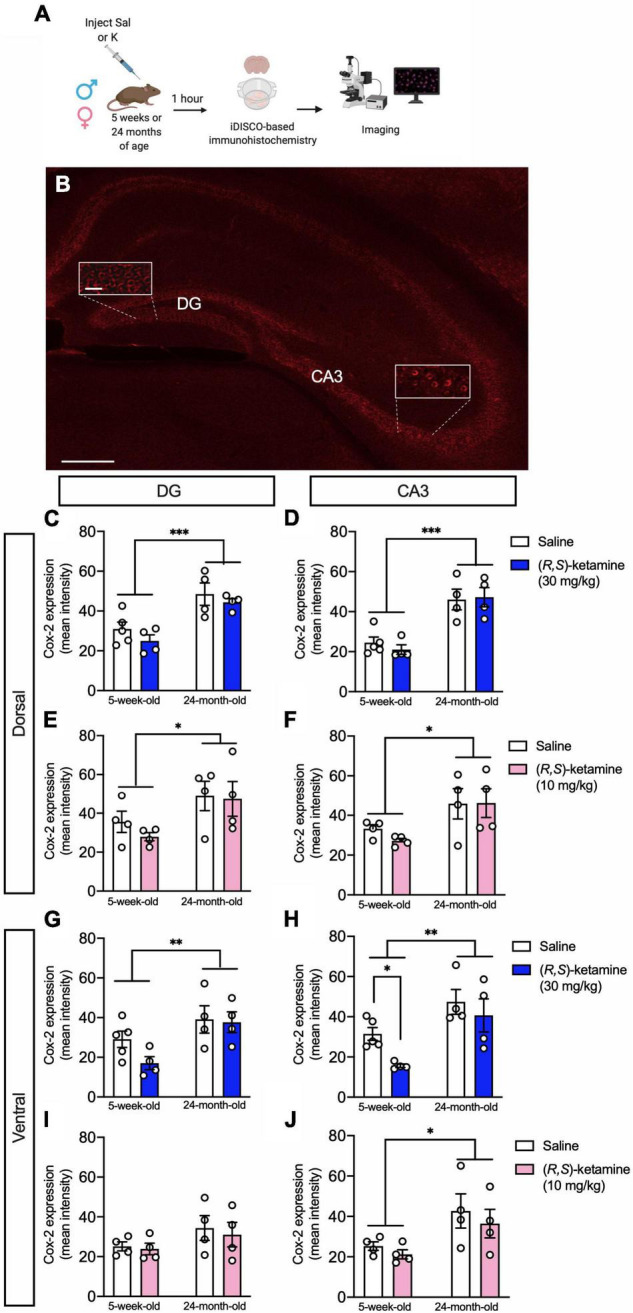
(*R,S*)-ketamine selectively decreases Cox-2 expression in ventral CA3 of adolescent male mice. **(A)** Experimental design. **(B)** Cox-2 expression in the hippocampus. Scale bar: 250 and 25 μm (inset). **(C,D)** Twenty-four-month-old male mice had increased Cox-2 expression in the dorsal DG and dorsal CA3 when compared with 5-week-old mice. (*R*,*S*)-ketamine did not impact Cox-2 expression. **(E,F)** Twenty-four-month-old female mice had increased Cox-2 expression in the dorsal DG and dorsal CA3 when compared with 5-week-old mice. (*R*,*S*)-ketamine did not impact Cox-2 expression in either region. **(G,H)** Twenty-four-month-old male mice had increased Cox-2 expression in the ventral DG and ventral CA3 when compared with 5-week-old mice. (*R*,*S*)-ketamine did not impact Cox-2 expression in the ventral DG. However, in 5-week-old male mice, (*R,S*)-ketamine decreased Cox-2 expression in ventral CA3. **(I)** All groups of female mice showed similar level of Cox-2 expression in the ventral DG. (*R*,*S*)-ketamine did not impact Cox-2 expression. **(J)** Twenty-four-month-old female mice had increased Cox-2 expression in vCA3 when compared to 5-week-old mice. (*R*,*S*)-ketamine did not impact Cox-2 expression. Error bars represent + SEM. *p < 0.05, ^**^p < 0.01, ^***^p < 0.001. Sal, saline; K, (*R*,*S*)-ketamine; DG, dentate gyrus; CA3, Cornu Ammonis region 3; iDISCO, immunolabeling-enabled three-dimensional imaging of solvent-cleared organs.

To assess whether the sex- and age-specific effects of (*R,S*)-ketamine on behavior could be associated with changes in inflammation, we quantified the expression of Cox-2, as proxy of inflammation (Galecki et al., 2012). Since we had previously found that (*R,S*)-ketamine administration changes neural activity in the DG and CA3 regions of the HPC (Mastrodonato et al., 2018), we measured Cox-2 expression across the HPC, specifically in the dorsal and ventral DG and CA3 subregions (Figures 4A,B). In the dorsal HPC, 24-month-old male mice had increased expression of Cox-2 in the DG and CA3 when compared with 5-week-old male mice [Age: *F*_(1,13)_ = 23.870, *p* = 0.0003; Age: *F*_(1,13)_ = 38.000, *p* < 0.0001, respectively] (Figures 4C,D). (*R*,*S*)-ketamine did not impact Cox-2 expression in either group [Drug: *F*_(1,13)_ = 1.790, *p* = 0.203; Drug: *F*_(1,13)_ = 0.086, *p* = 0.773, respectively]. Similarly, in the dorsal HPC, 24-month-old female mice had increased expression of Cox-2 in the DG and CA3 when compared with 5-week-old female mice [Age: *F*_(1,12)_ = 6.221, *p* = 0.028; Age: *F*_(1,12)_ = 8.507, *p* = 0.013, respectively] (Figures 4E,F). (*R*,*S*)-ketamine did not impact Cox-2 expression in either region [Drug: *F*_(1,12)_ = 0.487, *p* = 0.499; Drug: *F*_(1,12)_ = 0.281, *p* = 0.605, respectively].

In the ventral HPC, 24-month-old male mice had increased expression of Cox-2 in the DG and CA3 when compared with 5-week-old male mice [Age: *F*_(1,13)_ = 9.443, *p* = 0.009; Age: *F*_(1,12)_ = 15.520, *p* = 0.002, respectively] (Figures 4G,H). (*R*,*S*)-ketamine did not impact Cox-2 expression in either group in the ventral DG (vDG) [Drug: *F*_(1,13)_ = 1.821, *p* = 0.200]. However, 5-week-old male mice administered (*R,S*)-ketamine had decreased expression of Cox-2 in ventral CA3 (vCA3) when compared with saline-treated mice [Drug: *F*_(1,12)_ = 4.711, *p* = 0.049] (Figure 4H). (*R*,*S*)-ketamine did not impact Cox-2 expression in vCA3 in 24-month-old mice [Drug: *F*_(1,12)_ = 0.236, *p* = 0.636]. Five-week-old female mice had a similar level of Cox-2 expression in the vDG when compared to 24-month-old female mice [Age: *F*_(1,12)_ = 2.973, *p* = 0.110] (Figure 4I). Twenty-four-month-old female mice had increased expression of Cox-2 in vCA3 when compared with 5-week-old female mice [Age: *F*_(1,12)_ = 8.096, *p* = 0.015] (Figure 4J). (*R*,*S*)-ketamine did not impact Cox-2 expression in any of these groups [Drug: *F*_(1,12)_ = 0.819, *p* = 0.383]. These data suggest that (*R,S*)-ketamine effects might be partially due to the reduced expression of Cox-2 in vCA3 in 5-week-old male mice. Moreover, these findings are consistent with our previous data showing that vCA3 mediates (*R,S*)-ketamine efficacy against stress (Mastrodonato et al., 2018).

## Discussion

Here, we report for the first time that a single injection of (*R,S*)-ketamine, administered acutely, impacts behavior in adolescent, but not aged mice depending on sex and dose. Specifically, we found that (*R,S*)-ketamine administration reduced behavioral despair and perseverative behavior in female, but not male, mice and facilitated CFD in both sexes at specific doses.

In contrast to two different studies in male C57BL/6J and BALB/cJ mice, here, we found that (*R,S*)-ketamine administration (10 mg/kg) decreases behavioral despair (Zanos et al., 2016; Pham et al., 2018) in female mice. Although our data did not indicate antidepressant efficacy in male mice, our results may be specific to the drug doses, mouse strains, or behavioral paradigms we utilized. Moreover, it is possible that (*R,S*)-ketamine effects are more rapid in females than in males, similarly to what has been shown by a previous study (Franceschelli et al., 2015). Additional studies in humans have shown that (*R,S*)-ketamine has a rapid antidepressant effect in adolescents (Dwyer et al., 2017; Cullen et al., 2018; Zarrinnegar et al., 2019), but sex was not considered as biological variable. Previous works have also shown that testing mice of different genetic backgrounds in the same behavioral assays can lead to opposing conclusions, suggesting that a variety of mouse strains should be tested to determine (*R,S*)-ketamine efficacy (Sittig et al., 2016).

A recent study found that acute (*R,S*)-ketamine administration reduces locomotor activity and enhances avoidance behavior in adolescent male C57BL/6 mice (Shin et al., 2019). However, in this study, the authors administered (*R,S*)-ketamine at a different dose (15 mg/kg) than the one we used here, used mice of a different strain, and administered maternal separation early in life. Moreover, the effect of (*R,S*)-ketamine on behavioral despair, avoidance or compulsive behaviors were not investigated, and whether there was a sex- or age-specific effect was also not examined. In general the use of a different time of administration, sex, dose and/or mouse strain (McDougall et al., 2017; Shin et al., 2019) might explain why we could not find a significant (*R,S*)-ketamine effect in the LA experiment, and highlight the importance of these parameters in optimizing (*R,S*)-ketamine efficacy.

(*R,S*)-ketamine administration did not affect avoidance behavior 4 days following injection, consistent with previous studies showing the lack of an anxiolytic effect with (*R,S*)-ketamine (Autry et al., 2011; Brachman et al., 2016). However, (*R,S*)-ketamine decreased avoidance behavior in adolescent female mice 1 h following administration, suggesting that (*R,S*)-ketamine selectively affects avoidance behavior in a time-dependent manner. Future studies will assess additional time points for (*R,S*)-ketamine efficacy determining the most effective time windows of administration. Interestingly, we found that (*R,S*)-ketamine decreases compulsive behavior in adolescent female, but not male mice, suggesting the opportunity to treat compulsive-related disorders, such as obsessive compulsive disorder OCD (Rodriguez et al., 2013, 2015; Davis et al., 2021), specifically in a female population. Interestingly, while we found that (*R,S*)-ketamine decreases perseverative behavior only in adolescent females, it did not affect perseverative behavior when administered 1 h before MB testing, indicating that the time of administration is critical to observe an improvement in perseverative behavior.

Since impaired CFD is a symptom often observed in anxiety disorders, we assessed if the (*R,S*)-ketamine was effective against CFD in adolescent and aged mice (Altemus et al., 2014). Similar to adult mice (Mastrodonato et al., 2018), (*R,S*)-ketamine (30 mg/kg)-administered adolescent male mice and (*R,S*)-ketamine (10 mg/kg)-administered adolescent female mice discriminated faster than their saline-injected controls. Intriguingly, we also found that adolescent saline-injected male mice were impaired in CFD and showed fear generalization at 5 weeks of age (i.e., P35). These results are consistent with a previous study showing that there is an adolescent window of vulnerability (i.e., a “sensitive period”) in which adolescent male mice have impaired fear responses (Pattwell et al., 2016). Notably, adolescent saline-injected female mice did not show impaired CFD at P35, indicating that the neural circuits mediating fear behavior might be different between male and females at this age. These data are of critical importance because they indicate that (*R,S*)-ketamine could potentially be used to treat pathological fear generalization in anxiety disorders, which are prevalent during adolescence (Kessler et al., 2005). Moreover, these data suggest that the dose of (*R,S*)-ketamine will strongly determine its efficacy.

Interestingly, aged mice of both sexes could not discriminate (Wu et al., 2015), which is in accord with a previous study showing that aged mice are impaired in CFD when compared with young mice (Wu et al., 2015). (*R,S*)-ketamine did not facilitate CFD in aged mice. These results are also in line with recent studies showing that Spravato^®^ (esketamine) is less effective in older adults (i.e., > 60 years old) (Bahr et al., 2019) and that (*R,S*)-ketamine infusions are not effective in geriatric patients suffering from treatment-resistant depression (TRD) (Szymkowicz et al., 2014). These findings, therefore, indicate the necessity of testing different (*R,S*)-ketamine doses and protocols of administration in the geriatric population. Future work will be also needed to investigate a variety of doses and/or combined drug administration in aged mice.

Here, we evaluated whether inflammatory changes in the HPC could parallel the behavioral findings by examining Cox-2 expression 1 h following an injection of saline or (*R,S*)-ketamine. Interestingly, in addition to its antidepressant properties, (*R,S*)-ketamine has been shown to be an anti-inflammatory drug (Zanos et al., 2018; Verdonk et al., 2019) that regulates Cox-2 expression in several body tissues including the brain (Galecki et al., 2012; De Kock et al., 2013). We found that Cox-2 is significantly increased in the HPC of aged mice, which is in accord with mice and human data showing that several inflammatory markers increase with aging (Singh and Newman, 2011). Moreover, we found that (*R,S*)-ketamine selectively decreased Cox-2 expression in vCA3 of adolescent male mice. Ongoing experiments are clarifying whether Cox-2 levels are changed following different windows of administration of (*R,S*)-ketamine.

In summary, the present study highlights the necessity of age-, dose- and sex-specific pharmacological interventions to improve behavior. Future studies will investigate sex-specific brain circuits mechanisms that result in these behavioral effects with the ultimate aim to translate scientific data into practical applications that are effective for each sex.

## Limitations of the Study

There are some limitations in this study that could be addressed in future research. First, only the 1-h post-injection time point was analyzed rather than the 2 and 24 h-time points that are commonly used in preclinical and clinical studies (Zarate et al., 2006; Murrough et al., 2013; Zanos et al., 2016). However, there is considerable evidence that 1 h is an optimal time point. Numerous studies showed that (*R,S*)-ketamine is effective within 1 h of administration in adolescent, and aged subjects (Papolos et al., 2013; Srivastava et al., 2015; Zanos et al., 2016). In adolescent subjects, intranasal (*R,S*)-ketamine (30–120 mg) produces a significant therapeutic response within 1 h of administration that is sustained up to 72–96 h (Papolos et al., 2013). In aged subjects, (*R*,*S*)*-*ketamine (0.5 mg/kg) significantly reduces depressive symptoms (i.e., decreases the Hamilton depression rating scale (HAMD) scores of 65%) within 1 h of administration (Srivastava et al., 2015; George et al., 2017). The antidepressant response is sustained but not increased in the next 24 h post infusion (Srivastava et al., 2015). Finally, a recent study in adult mice has also shown that (*R,S*)-ketamine is effective at 1 h post-injection in adult mice (Zanos et al., 2016). Additionally, although short time points may be associated with side-effects, we did not see observe any side effects with respect to locomotion 1 h following injection. While locomotion assessment might not account for all the psychotomimetic effects, other studies have shown that they occur in less than 1-h post-administration (Papolos et al., 2013; Srivastava et al., 2015; Zanos et al., 2016).

Second, only a single injection of (*R,S*)-ketamine was tested in the elderly. Other studies have showed that repeated treatments resulted in higher likelihood of remission or longer time to relapse up to 6 months following infusions (George et al., 2017; Bryant et al., 2019), however, patients failed to sustain a response if the infusions were too far apart, therefore suggesting that repeated infusions may confer greater protection against depression, but they might need to be administered at greater frequency to be effective. Therefore, testing the effects of repeated injections might add to the findings of this analysis. This will be included in a new line of work.

Third, in this work we sought to investigate (*R,S*)-ketamine effects in adolescent and aged mice, and we did not include adult animals. However, we have previously published work showing the effects of varying doses of (*R,S*)-ketamine in adult male and female mice. In adult mice, we found that a single prophylactic injection of (*R,S*)-ketamine (30 mg/kg), but not 10 or 90 mg/kg, decreased behavioral despair and attenuated learned fear (Brachman et al., 2016; McGowan et al., 2017; Mastrodonato et al., 2018). In female mice, (*R,S*)-ketamine and its metabolite (2*R*,6*R*)-hydroxynorketamine [(2*R*,6*R*)-HNK] decreased behavioral despair, but did not attenuate learned fear (Chen et al., 2020). In female mice, (*R,S*)-ketamine was effective at a lower dose (10 mg/kg) than in male mice. Moreover, we showed that ovarian-derived hormones mediate the prophylactic actions of (*R,S*)-ketamine effects in female mice. These data further suggest that (*R,S*)-ketamine effects are age- and sex-specific and therefore emphasize the need for age- and sex-specific approaches to the prevention and treatment of psychiatric disorders.

Lastly, we did not found any amnesic effects following a single (*R,S*)-ketamine (30 mg/kg) administration in 129S6/SvEv mice (Brachman et al., 2016; Mastrodonato et al., 2018). Additionally, a recent paper found that a single injection of (*R,S*)-ketamine (30 mg/kg) administered 22 h after fear conditioning to C57BL/6 male mice, significantly facilitates CFD up to 2 weeks following injection (Asim et al., 2020). However, other rodent studies have shown that chronic injections of (*R,S*)-ketamine (30 mg/kg) induced learning and memory deficits (Neill et al., 2010; Tan et al., 2011). Therefore, it is not conclusive whether these findings will generalize to other mouse strains. We will investigate this in future work.

## Data Availability Statement

The original contributions presented in the study are included in the article/Supplementary Material, further inquiries can be directed to the corresponding author/s.

## Ethics Statement

The animal study was reviewed and approved by Institutional Animal Care and Use Committee (IACUC) at NYSPI.

## Author Contributions

AM and CD and JJM contributed to the conception and design of the work. AM, NK, and IP contributed to the acquisition of data. AM and CD contributed to the analysis and interpretation of data for the work, drafting the work and revising it critically for important intellectual content. AM, CD, NK, IP, JCM, and JJM approved the version of the manuscript to be published and agreed to be accountable for all aspects of the work in ensuring that questions related to the accuracy or integrity of any part of the work are appropriately investigated and resolved. All authors contributed to the article and approved the submitted version.

## Conflict of Interest

AM, IP, and CD were employed by Research Foundation for Mental Hygiene, Inc. CD and JCM are named on provisional patent applications for the prophylactic use of (*R,S*)-ketamine and other compounds against stress-related psychiatric disorders. The remaining authors declare that the research was conducted in the absence of any commercial or financial relationships that could be construed as a potential conflict of interest.

## Publisher’s Note

All claims expressed in this article are solely those of the authors and do not necessarily represent those of their affiliated organizations, or those of the publisher, the editors and the reviewers. Any product that may be evaluated in this article, or claim that may be made by its manufacturer, is not guaranteed or endorsed by the publisher.
